# The Regulatory Environment Surrounding Cannabis Medicines in the EU, the USA, and Australia

**DOI:** 10.3390/pharmaceutics17050635

**Published:** 2025-05-10

**Authors:** Claudia Jardim, M. Begoña Delgado-Charro

**Affiliations:** Department of Life Sciences, University of Bath, Bath BA2 7AY, UK; b.delgado-charro@bath.ac.uk

**Keywords:** medicinal cannabis, regulation, cannabidiol, tetrahydrocannabinol, Epidyolex™

## Abstract

**Background/Objectives**: Recent evidence supporting the medicinal use of cannabis has brought significant regulatory changes regarding its legal status, cultivation, and use. Several countries have adopted regulatory strategies enabling access to cannabis-based medicinal products; however, the regulation and availability of high-quality cannabis products differs globally. This work aimed to explore the regulatory pathways available for cannabis-based medicinal products, particularly those regulated as medicines, and establish the current landscape of those approved. **Methods**: The public repositories of the European Medicines Agency, U.S. Food and Drug Administration, and Therapeutic Goods Administration were searched. A consumer website, Amazon, was searched to provide illustrative examples of cannabis products readily available to consumers. Finally, clinical trial data were collected to evaluate trends in medicinal cannabis research. **Results**: Only Epidyolex™ has been approved by these three agencies. Whilst topical cannabinoid consumer products are popular, no topical cannabis-based medicines have been approved by regulators, despite being the focus of several clinical trials. There are few regulator-approved cannabis-based medicines available and, evidence supporting the therapeutic use of consumer products is very limited. A complex regulatory and legislative scenario hinders research on and development of cannabis-based medicines, leaving a market gap filled with unregulated products that are potentially misleading regarding their therapeutic claims.

## 1. Introduction

**Figure 1:**
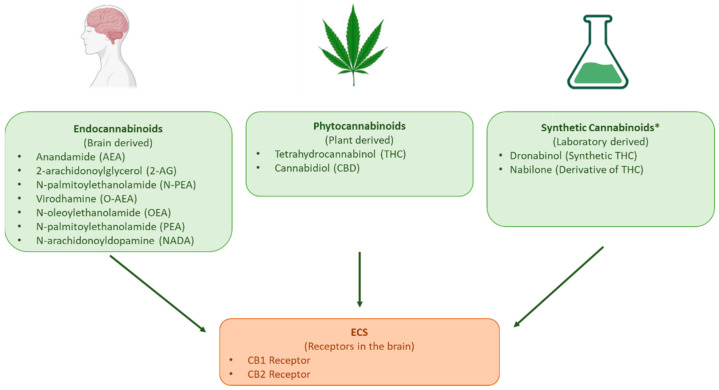
The three types of cannabinoids that interact with the endocannabinoid system. The phytocannabinoids THC and CBD and synthetic cannabinoids dronabinol and nabilone are included in CBMPs and in CBCPs. An exhaustive list of phytocannabinoids is available in reference [6]. Illustration created using https://BioRender.com (accessed 14 January 2025) based on information from [7]. * This list of synthetic cannabinoids does not include recreational synthetic cannabinoids as it is not part of the scope of this paper. The synthetic cannabinoids included in the figure are those used in CBMPs.

The potential therapeutic properties of cannabis have long been postulated [1]; yet, in the last century, many countries prohibited its cultivation, sale, and use [2,3]. The recent discovery of the endocannabinoid system (ECS) renewed interest in the medicinal use of the active compounds produced by *Cannabis sativa* [2,3,4]. Endocannabinoid’s effects are mediated through cannabinoid receptors 1 (CB1) and 2 (CB2). CB1 is primarily responsible for memory, mood, and the modulation of pain sensation, whilst CB2 is responsible for the anti-inflammatory and immunomodulatory effects of cannabinoids [5]. *Cannabis sativa* contains over 400 bioactive compounds [5], including more than 60 phytocannabinoids [6], which are structurally similar to endocannabinoids. The main phytocannabinoids that interact with the ECS and are commonly included in cannabis-based medicinal products (CBMPs) are tetrahydrocannabinol (THC) and cannabidiol (CBD) [4], though synthetic cannabinoids are also used (Figure 1) [7].

THC interaction with cannabinoid receptors causes the psychoactive effects associated with cannabis, whereas CBD interacts with the ECS and other neuromodulatory systems [5]. Given their psychotropic effects and potential for abuse, cannabinoids have been closely regulated; thus, a complex regulatory scenario evolved as their therapeutic potential was progressively supported by scientific evidence, alongside increasing public demand for the decriminalisation and legalisation of cannabis for medical use [2,3,4,8].

The 1961 UN Single Convention on Narcotic Drugs established an international regulatory framework by harmonising the principles for the control of narcotic drugs for medical and scientific use [9]. This framework does not avert signatory nations from allowing the use of cannabis for medical and scientific purposes within their territories [2,9], so divergencies in the regulation of CBMPs developed as countries implemented their own specific regulations [9]. It is believed that stringent and complex legislation hinders research into CBMPs [2,3,10].

The broad range of cannabinoid products expands from regulated and unregulated CBMPs to cannabinoid-based consumer products (CBCPs). This work classifies CBMPs into those approved and regulated as medicines (CBRMs) and those not approved/regulated as medicines (CBNMs). As with all medicines, CBRMs undergo rigorous quality, safety, and efficacy regulatory assessments (RAs) throughout their life cycle, by bodies such as the European Medicines Agency (EMA) or the Food and Drug Administration (FDA). Healthcare professionals prescribe CBRMs for indications and patients consistent with the outcome of clinical trials (CTs) [1]. In contrast, CBNMs might claim medicinal properties but are not medicines. Their regulatory scrutiny is less comprehensive, with them not being submitted to the same strict controls regarding quality control, efficacy, safety, and pharmacovigilance monitoring as CBRMs. The legal status of cannabis influences patients’ access to CBMPs, particularly in the case of CNRMs as previously reviewed [2,3,10]. It is for this reason that in the United States of America (USA), access to CBMPs differs amongst states [11]. In Australia, CBRMs are only available through prescription with the approval of the Therapeutic Goods Administration (TGA) [8,12].

Recent work [2,8] has extensively revised the status of medical cannabis in Brazil, Germany, Italy, the Netherlands, Canada, and Australia with a primary focus on the issues regarding regulation and patient access to CBNMs. Whilst this work also focuses on medicinal cannabis, its primary focus has been CBRMs and hence, their regulation by the EMA, FDA, and TGA as representatives of three main regulatory areas.

Regarding CBCPs, there is a growing interest in topical cannabinoids specifically [10,13]. These products are often accessible to consumers for cosmetic use, advertised as “health and well-being” products, and are merely subject to the relevant cosmetic product regulations [10]. Cannabinoids are among the novel ingredients incorporated by the cosmetics industry [11], and the growing interest in topical cannabinoids has been shown by the numerous CBCPs applied to the skin as creams, ointments, gels, and patches. However, there are concerns that the advertising of some of these topical CBCPs may mislead customers about alleged dermatological properties [10]. For these reasons, this report explored which (if any) clinical evidence underlies these “dermatological properties”. To do this, the current range of topical CBMPs and their potential future landscape (as anticipated from clinical trials on topical CBMPs) were explored. Other cannabis derived products including dietary supplements and novel foods are beyond the scope of this work as they have been recently and extensively reviewed, see for example [14,15,16,17,18,19].

This work aimed to (i) provide a perspective on the current regulatory framework controlling the development and approval of cannabis-based regulated medicines (CBRMs) in Australia, the EU, and the USA; (ii) explore the range of CBRMs currently available for patients; (iii) explore the range of potential future therapeutic indications for CBRMs as informed by clinical trial research; and (iv) provide an illustrative example of available online CBCPs. Note that business analysis, market availability and access, policy enforcement, and clinical (safety/efficacy) analysis aspects are beyond the scope of this work.

## 2. Materials and Methods

### 2.1. Data Mining

The prior literature and regulatory agencies’ websites were searched to establish the regulatory framework for CBMPs in different countries, after which datasets were generated. (i) Regulatory repositories were used to gather information on CBMPs either marketed or undergoing marketing authorisation assessment and to document the road-to-market for Epidyolex™ [20,21,22,23,24]. (ii) A popular consumer website, Amazon (https://www.amazon.com/, last accessed on 31 December 2024; Amazon.com, Inc. Seattle, WA, USA), was used to provide illustrative examples of CBCPs with alleged health and well-being properties that are freely available to consumers online [25]. Google Trends was initially searched to draw out the countries with high online interest in CBCPs and the product keywords that were often searched for. Amazon was then used to search for available products, as it is the largest global online retailer, and the search could be replicated for several countries [26]. The purpose of searching Amazon was to exemplify the type of information and products that a consumer could readily find with a simple random search and not to conduct an exhaustive analysis of the online market for CBCPs.

Finally, (iii) a database (AdisInsight) available through the University of Bath library was used to collect information about CTs conducted with cannabinoids (https://adisinsight.springer.com/, last accessed on 31 December 2024) [27]. AdisInsight was used as it offers detailed information on drug development, including regulatory milestones, which is a focus of this report. This database integrates data on clinical trial outcomes as well as regulatory decisions. Additionally, the database includes clinical trial data from a wide range of countries and regions that were essential for the scope of this research.

### 2.2. CBMPs Regulatory Framework

Web of Science was searched using the following keywords and Medical Subject Heading (MeSH) terms: (1) medicinal cannabis OR cannabi* OR tetrahydrocannabi* OR THC OR dronabinol OR nabiximol and (2) Regulat* OR regulatory framework. Web of Science was selected as the database of choice as it produced the highest number of results, including outputs gathered from alternative databases (Google Scholar and PubMed). Articles published in English from 2018 to 2024 were included as in 2018, Epidyolex became the first CBMP to be approved by the FDA [20]. Articles discussing recreational cannabis were excluded. This search produced a total of 1994 articles. The titles and abstracts were reviewed to determine their relevance to the research question (see Table S1). Following this, a total of 104 articles were deemed relevant for this research.

Additionally, information about the regulation of CBMPs was gathered from the EMA, FDA, and TGA public repositories as explained in Section 2.3.

### 2.3. CBRM Dataset

The EMA download tool (https://www.ema.europa.eu/en/medicines/download-medicine-data, last accessed on 31 December 2024) was used to find CBRMs available in the EU [22] through the centralised procedure. This path, followed by most innovative medicines, is compulsory for some therapeutic indications and for orphan medicines [28]. The EMA download tool lists all the approved medicines, withdrawn applications for new marketing authorizations, refused marketing authorizations, and legal status for over 2000 medicines as an Excel table (Table S2)

Results were filtered to human medicines and to “cannabidiol”, “nabiximol”, “tetrahydrocannabinol”, “dronabinol”, and “cannabis” as active pharmaceutical ingredients (APIs). For each record, the authorisation and orphan designation status, indications, and route of administration (ROA) were collected. Documents such as paediatric investigation plans (PIPs) and European Public Assessment Reports (EPARs) were collected to gather information about CTs (study design and results), safety and adverse reactions, benefit-risk assessments, and regulatory decisions.

The Drugs@FDA database [20] (https://www.accessdata.fda.gov/scripts/cder/daf/index.cfm, last accessed on 31 December 2024) was used to find CBRMs available in the USA, by searching for the same criteria as above. Documents such as FDA reviews, letters, and product labels were scrutinised to gather product-specific information.

The Australian Register of Therapeutic Goods (ARTG) (https://www.tga.gov.au/products/australian-register-therapeutic-goods-artg, last accessed on 31 December 2024) was used to find CBRMs available in Australia [24]. The search included products described as “medicines” containing “dronabinol”, “tetrahydrocannabinol”, “nabiximol”, “cannabidiol”, “hemp”, or “nabilone” [24]. Information about APIs, ARTG start dates (the date the product was added to the ARTG), marketing status, and indication were collected. The permitted indications and specific indications were noted for export-only (EO) medicines. The EO medicines dataset is available as Table S3 of the Supplementary Information. TGA documents such as public summaries, patient information leaflets, and product information were scrutinised to gather more information on the specific products and their road-to-market journeys. Finally, the “medicinal cannabis hub” (last accessed in August 2024) of the TGA was searched for information about regulation and control of Australian CBNMs [29].

The search term “cannabidiol” on the EMA webpage provided many results relating to orphan designation. Additional searches with this keyword were conducted across the three agencies’ registers for orphan designation products [30,31,32]. Finally, regulatory information regarding the road-to-market for Epidyolex™ was gathered to construct a timeline of key regulatory events. For this, the EMA-EPARs, Australian public assessment reports (AusPAR), and FDA product labels were used.

Table S4 presents the exclusion/inclusion criteria followed when gathering regulatory information and data.

### 2.4. CBCP Dataset

The Google Trends platform was used to assess consumer interest in CBD products. The search topic “cannabidiol” was searched, as on Google Trends, search topics capture a broader range of results than search terms [33]. The search did not have enough data for Google Trends to provide results; therefore, the search term “CBD” was used to query search requests in the shopping category worldwide, from August 2019 to December 2024 [33]. Additionally, top-related topics and queries to the search were extracted and these included “Cannabidiol- medication”, “oil-topic”, “cbd oil”, and “cbd oil buy”. These top-related topics indicate topics that users also searched for along with our original search terms (Table S5) [33].

Google Trends analyses a sample of search data to measure the popularity of search queries over time and across different regions. It normalises the data by comparing searches to the total search volume, thus displaying results as a relative index rather than absolute values [34].

Whilst use of Google Trends (see Section 2.6) presents limitations, Narayanan et al. 2020 provides a guideline for its use to provide an illustrative example of the public interest in cannabis-based products that are easily available to consumers online [34,35].

Next, the domains for Australia and New Zealand, the USA, and France of the consumer website Amazon (www.amazon.com) were searched (accessed on 12 December 2024) using “CBD oil”, “topical CBD”, and “CBD cream” as keywords, informed by the Google Trends platform search. Amazon is the largest global online retailer, and the search could be replicated for the countries highlighted by the Google Trends results [26].

The purpose of the search conducted through Google Trends and Amazon was only to illustrate the type of information and products that a consumer could easily find in one random search and neither to perform an exhaustive analysis of the online market for CBCPs nor to produce results for either economic or policy analysis. This search aimed to find topical cannabinoid products and included oils, which may either be directly topically applied to the skin or incorporated into topical products. The results were limited to the categories “cosmetics” and “health products”, and products that were not topical cannabinoid-based were later removed. The “indications”, “ingredients”, and “marketing authorisation” of these readily available CBCPs were collected where available.

### 2.5. Clinical Trials with Cannabinoid-Based Products

The AdisInsight database (https://adisinsight.springer.com/, last accessed on 31 December 2024) provided relevant CT data by filtering the drug class to “cannabinoids” (raw data in Table S6). Trials not involving cannabinoids (plant-derived or synthetic) as the primary drug and using endocannabinoid mimetics were removed from the final dataset. The curated data were exported to Excel and data on the primary sponsor, trial phase, indications, trial status, investigated cannabinoids, and ROA were extracted. Inclusion and exclusion criteria were applied to all outputs produced from AdisInsight (Table S7).

### 2.6. Data Analysis and Study Limitations

Data extracted (see Datasheets in Supplementary Materials) were further classified and analysed using Microsoft Excel. This involved manual data mining, visual inspection, and processing, so some data and information might have been missed by error only, as no relevant information regarding the regulation of CBRMs by the EMA, FDA, and TGA was deliberately omitted. The data presented in this review provide a “December 2024 snapshot” of regulatory processes, CTs, and product landscape without further updates.

The authors acknowledge the following limitations of the study: (a) Search data available through Google Trends and Amazon only reflects those with internet access, leading to coverage bias [35]. (b) The data extracted from Amazon is impacted by marketing strategies and the algorithm used by businesses on the consumer site, which could influence the results produced by our searches [36]. Given these limitations, the data are presented here with exemplary purposes only, i.e., to illustrate products and labelling that might be found by an online consumer in a random search. Importantly, search results may vary with several factors as the geographical location, chronological time of the search, and algorithm effects do not necessarily reflect the interests of non-online active consumers [35].

## 3. Results

### 3.1. The Cannabis-Based Medicinal Product Landscape

This work gathered information on the current regulatory environment surrounding CBRMs and explored the landscape as informed by three (EMA, FDA, and TGA) key regulatory agencies. Table 1 shows records of regulatory events and approved CBRMs. Only Epidyolex™ was recommended for authorization by the three agencies. Sativex™ went through the EU National Competent Authorities and the TGA. Dronabinol-only products have been approved by the FDA, including four generic products now discontinued. The EMA and FDA have granted orphan drug status/designation to 51 cannabidiol products (Table S2), with one orphan medicine having proceeded through the EMA and TGA and two through the FDA (Table 1).

**Figure 2:**
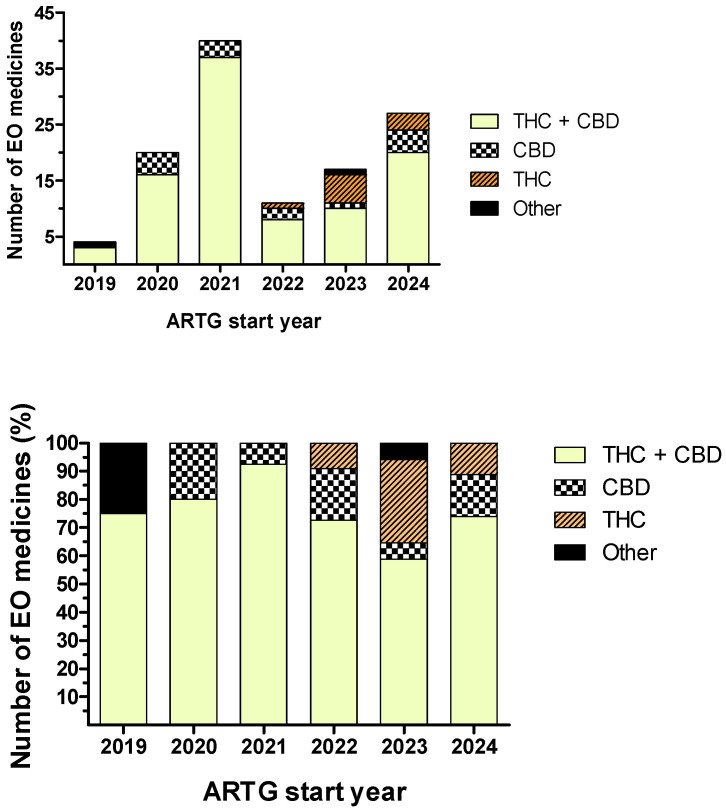
Distribution of ARTG—export only-medicines according to their ARTG start date and active ingredients. **Top** panel: value indicates the number of products in each category. **Bottom** panel: percentage of each product category with respect to the total number of products added in the corresponding year (last accessed on 10 December 2024).

Further to the four TGA products in Table 1, the ARTG repository listed 116 EO medicines (Table S3; Figure 2), most involving CBD-THC combinations (*n* = 94) of which sponsors often provided multiple CBD:THC ratios (*n* = 30). Other products contained a single API—cannabidiol (*n* = 12) or tetrahydrocannabinol (*n* = 7)—and rare combinations—tetrahydrocannabinol with dronabinol (*n* = 1) and cannabidiol with melatonin (*n* = 1). Oral liquids, mostly oils (*n* = 92), were preferred compared to herbs dried for oral administration (*n* = 5) and inhalation (*n* = 19), sublingual wafers (*n* = 4), buccal solutions (*n* = 1), and buccal sprays (*n* = 1). Figure 2 shows the distribution of EO medicines according to their ARTG start date and APIs. No “EO medicines” were found before 2019, and the 2021 peak in “ARTG start dates” (*n* = 40) has continuously decreased until 2023 (*n* = 17). CBD:THC combination products were the most numerous in 2021 (93%) but represented only 59% in 2023. Concomitantly, products with tetrahydrocannabinol became more numerous since 2022.

### 3.2. The Cannabis-Based Consumer Product Landscape

**Figure 3:**
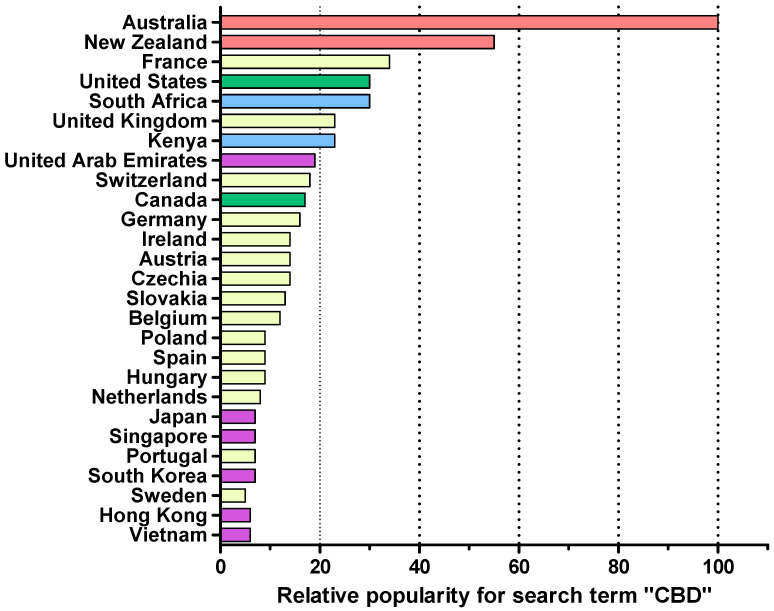
Relative popularity of the term “CBD” under the shopping category from 2018 to December 2024 across different countries. The popularity in each location is determined as a fraction with respect to all other searches at the specific location. A value of 100 is assigned to the country with highest popularity. Note that popularity represents the frequency of the query with respect to other queries, not the absolute number of queries. Data generated using Google Trends [33], last accessed on December 2024. Please refer to Section 2.6 regarding limitations associated with the use of Google Trends. The colours of the bars divide the countries into their respective regions.

The illustrative search on the Google Trends platform identified three main geographical regions expressing interest in the topic of cannabis (Figure 3). The top related queries to the search terms narrowed down the type of cannabis products that consumers were searching for and revealed particular interest in CBD oil.

**Figure 4:**
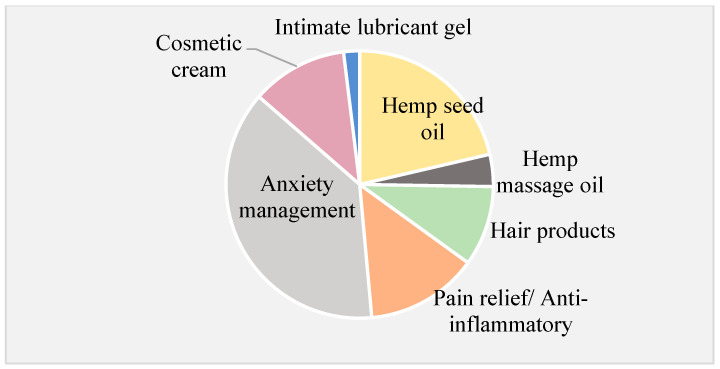
Illustrative example of the suggested indications and uses proposed for topical cannabinoid-based consumer products identified online as readily available to consumers. These results are shown for exemplifying purposes only. Data collected from www.amazon.com [25], last accessed on December 2024. Please refer to Section 2.6 regarding limitations associated with this study.

Searching for “CBD” on the Amazon website in the Australia, New Zealand, and US domains revealed that less than 1% of the products displayed the phrase “cannabinoid” on their ingredient label. None of the products listed on the Australia and New Zealand website were available for purchase. In contrast, 63% of the 1000 products displayed on the French Amazon website were labelled as “cannabinoid-based”. The French Amazon website contained the largest number (*n* = 91) of topical CBCPs available for purchase. Less than half of the CBCPs on the American and French websites provided ingredient lists. Most topical CBCPs were marketed as “hemp oil” or “CBD oil”, often being suggested for the management of anxiety, for pain relief, or as anti-inflammatory agents (Figure 4). A large proportion of hemp seed oil products were labelled as nutritional supplements for “general well-being”.

### 3.3. Clinical Trials Involving Cannabinoids

The AdisInsight database provided 666 clinical trials involving cannabinoids (Table S6). Following data curation (see Methods and the inclusion and exclusion criteria in Table S7), 658 clinical trials remained in the dataset. Table 2 and Table 3 list the status and indications for the CTs extracted. A total of 307 CTs were completed, mostly for epilepsy and multiple sclerosis (Table 2), and 70 trials were either discontinued, suspended, withdrawn, or displayed unknown trial status (Table 3). The most prevalent reasons provided for CTs failure were low recruitment numbers and a lack of funding for study continuation. Twenty-four CTs focused on topical cannabinoids of which nine were completed, two were withdrawn, and seven CTs were in planning/recruitment stages (Table 4). Most were phase I/II CTs, and the most popular indication was epidermolysis bullosa.

## 4. Discussion

### 4.1. Regulatory Framework for Medicinal Cannabis Products

There is no EU framework specific to CBMPs. A 2023 EMA document clarified that these products must comply with the same requirements as any other medicinal product, as described by EU pharmaceutical law (Article 1(2) of Directive 2001/83/EC and Regulation (EC) 726/2004 for centrally authorised products), and thus must obtain marketing authorisation (MA) from a competent authority before reaching the EU market [38]. Nevertheless, developers are advised to familiarise themselves with potential national requirements as the conditions for CBRM distribution and patients’ access across the EU are not harmonised [38]. In the EU, a medicinal product may be authorised either by the European Commission, after assessment through the EMA’s centralised procedure, or by National Competent Authorities (NCAs), through mutual recognition, decentralised procedures, or national procedures [28]. Table 1 shows these pathways for Epydiolex, an orphan product that followed the centralised procedure, and a range of products approved by National Competent Authorities [4].

Depending on its composition, a CBRM may be considered an herbal medicine product (HMP) under EU legislation. Developers should thus follow procedures overseen by National Competent Authorities [39]. Three regulatory pathways enable the marketing of HMPs in EU Member States: “traditional use registration”, “well-established use MA”, and “stand-alone/mixed application” [39]. HMPs containing new active substances of herbal or synthetic origin (including cannabis-derived substances/synthetic cannabinoids) do not meet the requirements for “well-established medicinal use” or “traditional use” paths and must comply with the general provisions for MA [39]. The EMA provides HMP guidelines and has published a compilation of cannabis-related terms and definitions for clarification [39]. To be considered an HMP, the active ingredients must exclusively contain, “one or more herbal preparations, or one or more such herbal substances in combination with one or more such herbal preparations” [40]. APIs contained in cannabis-derived HMPs may be an isolated constituent, herbal substance(s), herbal preparation(s), or a combination of the above [40]. Herbal substances derived from cannabis that fit the ‘herbal substance’ definition in Direction 2001/83/EC and the Ph. Eur. Monograph 1433 include flowers (*Cannabis flos*) and resin (*Cannabis resina*). A Ph. Eur. monograph for ‘*Cannabis flos*’ or derived preparations or constituents is not available, but the national Danish and German pharmacopoeias provide monographs [40]. The recent Committee on Herbal Medicinal Products (HPMC) call for scientific data suggests that steps are being taken to support the development of EU herbal monographs and/or EU list entries regarding *Cannabis sativa* L. [38].

There is no common EU regulatory framework for magistral and officinal preparations containing cannabinoids as these types of preparations are exempt from MA [4]. These preparations are controlled at national level according to each country’s specific regulations and frameworks on narcotics and individual prescriptions [4,41]. Regarding CBNMs, cultural and legislative differences towards cannabis leads to inconsistent patients’ access across EU member states [4,8]. For example, in the Netherlands, doctors have been able to prescribe medicinal cannabis since 2003 [3,4]. In contrast, French regulation of medicinal cannabis is stringent, and CBNMs are only prescribed to patients with specific medical conditions once all other medications have failed [4].

The USA Controlled Substances Act (CSA) classifies cannabis as a Schedule I controlled substance at federal level, thus making it illegal for most purposes. The Agriculture Improvement Act of 2018 (Farm Bill 2018) modified the cannabis status under the CSA [42]. This bill defined hemp as “*Cannabis sativa* plant and derivatives or extracts of the plant with no more than 0.3% by dry weight of THC” and removed hemp from the definition of marijuana so that it was no longer a controlled substance under federal law [42]. This reform aimed to facilitate research on substances meeting the newly defined ‘hemp’ category, including CBD, and to fasten development of medicines containing hemp [43].

In the US, medicines must be approved centrally through the FDA before reaching the market [42]. Most drug products must either (i) receive premarket approval for a New Drug Application (NDA) or Abbreviated New Drug Application (ANDA), or (ii), for certain over-the-counter non-prescription drugs, meet the requirements in the Federal Food, Drug and Cosmetic Act (FD&C Act) for marketing without an approved NDA or ANDA [42]. According to Section 201(g) of the FD&C Act, “any product (including one that contains cannabis or cannabis-derived compounds) which is marketed with a claim of therapeutic benefit, or with any other disease-related claim, is considered a drug” [42]. Thus, CBRMs are subject to the same FDA controls and requirements as any other drug product [42]. Acknowledging the complexities surrounding cannabis products, an FDA webpage provides developers and consumers with additional information on the regulation of cannabis and cannabis-derived products [42,44]. Additionally, CBMP developers have access to guidance in the Botanical Drug Development Guidance for Industry, as well as the Cannabis and Cannabis-Derived Compounds: Quality Considerations for Clinical Research [43,45]. Drug products containing fully synthetic versions of substances (e.g., dronabinol) and cannabis-related compounds are regulated like any other fully synthetic drug [43].

The FDA regulates cannabis-based products in accordance with federal law, but state regulations governing cannabinoid products differ [1]. Some states have removed state restrictions on the medical use of cannabis and its derivatives and set up programmes enabling legal use of CBNMs without FDA approval [1,42]. State medicinal cannabis programmes may diverge regarding which indications qualify for treatment, the types of products available to patients, and the standards of product quality and safety [1]. Notably, CBNMs cannot be prescribed but “recommended” by healthcare providers in these states [1]. The lack of harmonisation at the federal and state levels can impact patient access to CBNMs and create inconsistencies in the products’ standard and quality.

In Australia, as any other medicinal product, CBRMs follow a centralised approval process through the TGA, requiring evidence of quality, safety, and efficacy, clearly defined APIs, and appropriate packaging and labelling, before inclusion in the ARTG and legal market entry [12]. Whilst the TGA regulates access to CBRMs, most medicinal cannabis products in Australia are CBNMs, i.e., not assessed for safety, efficacy, and quality and, therefore, not registered on the ARTG [29]. Medical use of CBNMs is legal in Australia under certain conditions, and supply is authorised by the TGA [29]. Different to the other agencies, the TGA provides a dedicated CBNM “hub”, offering information and explaining access pathways [29]. CBNMs can be accessed via unapproved medicine pathways: the “special access scheme” (SAS) and the “authorized prescriber scheme” [29]. The SAS is intended for clinical circumstances in which all approved medicines have been tried, and access to an unapproved medicine is required [29]. The SAS grants authority to a doctor to supply an unapproved medicine/medical device to treat either life-threatening conditions (SAS-Category A) or serious but not life-threatening conditions (SAS-Category B) [29]. By July 2019, the TGA had approved over 11,000 SAS applications of medicinal cannabis for conditions such as neuropathic pain and refractory paediatric epilepsy [29]. Through the authorised prescriber scheme, a doctor may access unapproved cannabis on behalf of the patient [28].

All three agencies’ approaches towards CBRMs are similar, treating these products as any other regulated medicine. Notable differences include the EU network structure and national competencies regarding HMPs, the central-level (FDA/EMA) versus state-/country-level regulation of CBNMs in the USA/EU, and the access pathways to CBNMs through the TGA.

### 4.2. The CBRM Landscape

The CBRMs in Table 1 undergo regulatory assessment and are subject to pharmacovigilance procedures [2]. Despite potential inter-agency differences regarding the documentation of quality, safety, and efficacy by sponsors, this regulatory process sets CBRMs apart from CBNMs and CBCPs. Regrettably, this difference is not always appreciated by the public [13].

The FDA recommended the approval of four CBRMs, one cannabis-derived drug product—Epidyolex™ (cannabidiol, GW Pharmaceuticals, United Kingdom)—and three synthetic, cannabis-related, prescription-only, drug products—Marinol™ (dronabinol, Solvay Pharmaceuticals, Belgium), Syndros™ (dronabinol, Benuvia Therapeutics Inc., United States), and Cesamet™ (nabilone, Meda Pharmaceuticals Inc., United States, Canada and United Kingdom). Four of the five dronabinol generics approved are currently discontinued [41]. Only Epidyolex™ went through the EMA’s centralised procedure, whilst numerous Sativex™-type products went through national procedures. Four products were approved for the Australian market: Epidyolex™, Sativex™, and two hemp-seed medicines.

Epidyolex™, a CBD-only medicine for the treatment of rare forms of epilepsy (Table 1) was recommended for approval by the three agencies. The product was granted orphan drug (OD) designation for the adjunctive treatment of Lennox–Gastaut and Dravet Syndrome by the three agencies and, additionally, by the EMA for tuberous sclerosis complex [21,23]. Epidyolex™ followed the FDA and TGA priority pathways that support faster access for patients to innovative medicines [21,23]. Epidyolex™ efficacy and safety for patients over 2 years of age was tested through several randomised, double-blind, placebo-controlled, parallel-group studies and its benefit–risk ratio demonstrated [21,23]. Given the lack of data on pregnant, lactating, or breast-feeding women, Epidyolex™ should not be used by these populations unless the potential benefits to the mother clearly outweigh the potential risks to the foetus [21,23,46]. Unfortunately, participation of pregnant women in CTs was further challenged by ethical concerns surrounding cannabis use. The post-marketing observational cohort study, part of the EMA-approved pharmacovigilance plan presented, will only include European patients, as the sponsor has justified its plans to extrapolate findings to Australian patients [46].

Given the alleged interest in and demand for CBMPs, the unexpectedly small number of CBRMs is potentially explained by the alternatives provided by CBNMs. CBNMs reach the market more easily and with at a lower cost, given their reduced level of regulatory scrutiny and monitoring. Indeed, even when CBNMs are more strictly regulated for their cultivation, import, and consistency, as is the case of the Dutch market [3,8], these products do not require safety and efficacy trials to reach patients. Quality testing safeguards the public by verifying active ingredient contents and the absence of toxic impurities; however, it does not guarantee bioequivalence. Yet, it is well known that products with same active strength may provide different rates and extents of absorption (i.e., effective dose) and effects. Despite the significantly limited knowledge about CBNMs’ safety and efficacy, these products capture sufficient trust among public and healthcare professionals as to be prescribed, so there is little incentive for investors to support development of CBRMs. The latter will incur higher development costs and will encounter a reduced market given what could be considered unfair market competition [8]. Whilst many medicines face competition from generics and biosimilars, the entry of these products into the market is strictly regulated. Paradoxically, some healthcare professionals hesitate prescribing CBNMs because of the perceived stigma and lack of knowledge regarding their quality, safety, and efficacy [2,3,8], precisely what regulated medicines provide. Finally, it could be argued that most of the strategies proposed for safer use of CBNMs [3,8] are addressed by the development of CBRMs. This complex scenario is, perhaps, quite specific to cannabis products.

Two medicines approved for the Australian market fall into the licence category LI (listed medicine) and two fall into RE (registered medicine). Listed medicines are assessed for quality and safety, but not for efficacy [31]. Thus, the pain relief and plant-based omega (Table 1) indications were selected from a list of pre-approved “permitted indications”. In contrast, registered medicines are considered ‘higher risk’, and Sativex™ and Epidyolex™ were fully assessed for quality, safety, and efficacy through CTs [46]. It is unclear whether Australian patients and healthcare workers are aware of these differences and whether this impacts prescription patterns and/or adherence to treatments.

OD designation was relatively commonly granted by regulatory agencies (Table 1). OD designation is assigned to medicines in development that intend to treat, prevent, or diagnose a life-threatening disease with low prevalence amongst the general population [47]. OD designation provides a series of incentives and benefits for sponsors to aid the development of orphan medicines [47]. As mentioned above, CBRMs must meet the same requirements as other medicines; however, their development is hindered by additional legislative barriers and enhanced market competition with CBNMs. By targeting OD status, sponsors could receive financial incentives (reduced regulatory fees, extended market exclusivity, and tax credits), increased regulatory support through development, and enhanced access to patients through the demonstration of safety and efficacy for a specific indication [47,48]. OD benefits are particularly beneficial for small enterprises and universities, which constitute the majority of CBRM sponsors.

The large difference between the number of ARTG products approved for the Australian market (*n* = 4) and EO products (*n* = 116) (Table 1 and Table S3, Figure 2) suggests that the manufacturing of cannabinoid-based medicines for third countries is a preferred activity by Australian sponsors. Unlike the products in Table 1, most EO records (*n* = 96) indicated “no permitted/specific indications” only, and a small number (*n* = 20) listed specific indications.

Often (*n* = 11), the specific indications employed language such as “there is growing evidence…”, suggesting a lack of CTs substantiating the therapeutic benefit of the product for the indications listed. EO medicines cannot be supplied in Australia (Section 26, 1989 Therapeutic Goods Act) but must be ARTG-listed to ensure compliance with Good Manufacturing Processes (GMPs), like products supplied in Australia. EO medicines must be safe for their intended purpose(s) of use, have acceptable presentation, comply with required quality and safety standards, and be subjected to advertising provisions. The TGA provides a Certificate of Pharmaceutical Product for EO medicines that is issued under the WHO Certification Scheme and is accompanied by a mandatory Schedule 1—Formulation and an optional Schedule 2—Manufacturers [49]. EO medicines may have to comply with third-country regulations to either enter the market or be used in CTs; however, the ARTG does not report their final use and destination.

### 4.3. The “Well-Being” CBCP Landscape

The results of the illustrative search using the Google Trends platform confirmed the consumers’ interest in CBCPs, and the subsequent analysis of this market highlighted the differences in accessibility and the variety of topical CPCPs readily available to consumers. In the search conducted, CBCP products fell within the category of cosmetics and should be regulated as such, yet the description of some CBCPs (Figure 4) could mislead consumers and patients to perceive them as medicines. Topical CBCPs were often advertised for relief of anxiety and pain and as anti-inflammatories. No products were available for purchase from the Australia and New Zealand site as, from 2015, CBD products are not legally available without a prescription [50].

The results reported in Figure 3 and Figure 4 are provided as an example about what an online active consumer could find in a random search. Indeed, the outcome of such a search would differ for several reasons [35]. This example served to illustrate, graphically, the concerns regarding the mislabelling of therapeutic properties [51,52,53] and the inconsistent and poor labelling of CBCPs [54,55] previously reported. In this work, scrutiny of ingredient lists identified “hemp seed oil” as the most common main component. This oil is extracted from the seeds of *Cannabis sativa* and should contain only trace amounts of active phytocannabinoids, adding to concerns about the legitimacy of “health claims” by CBCPs [23]. The marketing of non-FDA approved CBCPs claiming medical benefits is forbidden [50], yet a study in CBCPs available in North Carolina found that retailers often advertised them as legitimate options for health purposes [56]. The FDA has issued several warning letters to companies regarding the illegal marketing of unapproved CBD products claiming to treat medical conditions; see, for example, ref. [57]. The loose regulation of CBCPs has potential for patient harm as consumers enticed by unsubstantiated health-related claims in marketing materials and labels may forgo evidence-based medical interventions [13,58]. The lack of stringent regulatory oversight allows CBCPs to be marketed with misleading claims, potentially fostering false expectations and delaying patients’ access to evidence-based treatments. This raises ethical and public health concerns, as reliance on unproven products may not only exacerbate health conditions but also undermine trust in conventional medical interventions. Consumers require education to distinguish between CBCPs and CBRMs in order to critically appraise information and social media perceptions regarding the health benefits of CBCPs [59].

### 4.4. Future Trends in CBMPs: Ongoing Clinical Trials

The lack of well-designed and powered clinical trials on the use of medicinal cannabis is considered one of the main challenges regarding these products [2] and critical to answer questions regarding long-term safety in higher risk populations, drug interactions, the impact of route of administration and dosage form, and pharmacogenetics [3]. Together with pharmacovigilance, risk assessment plans, and real-world evidence studies, CTs constitute the main tools to monitor the safety and efficacy of medicinal products and ensure that their benefit/risk ratio is positive for diverse patients’ population. CTs involving cannabinoid actives were gathered to assess the activity and future trends relating to CBRMs (Table 2, Table 3 and Table 4). Most CTs corresponded to phase I and II, with the main reason for CT failure being a lack of funding, which was unsurprising as sponsors were often either small enterprises or research groups. Consistently with Table 1, some CTs addressed orphan indications and hence addressed unmet medical needs. This illustrates another of the dilemmas and challenges in this area. On the one side, restriction of the use of CBNMs has been proposed for patients with debilitating and life-threatening diseases without medical alternatives, on the basis that the benefit/risk ratio would be, potentially, more positive for these patients [2]. On the other hand, it could be argued that this approach may potentially harm these patients both directly, by exposing them to products whose quality, safety, and efficacy have not been established, and in the longer term, by disincentivizing the robust research and development of orphan regulated medicines, a path which is well supported by regulators. Regarding completed CTs (Table 2), the most addressed indications were aligned to those already covered (epilepsy, pain, nausea, and vomiting) by current products.

The illustrative example of the consumer market highlighted the prevalence of topical CBCPs; thus, exploring trends in topical products being investigated as medicines was pertinent (Table 4). Completed phase I and II CTs with topical cannabinoids focused on dermatological indications such as atopic dermatitis and epidermolysis bullosa (potentially an OD product). Regarding pain management, no completed CTs were found though two were actively recruiting. Atopic dermatitis and psoriasis are dermatological conditions associated with poor skin barrier function, and, together with acne, they were the indications that could have been more “loosely associated with skin well-being claims”. For this reason, the status of CTs on these indications was of particular interest (Table 4). In the case of atopic dermatitis and acne, Botanix Pharmaceuticals Limited reported successful results of its randomised, double-blind, placebo-controlled phase 1b patient study [27]. But no further developments were reported. Regarding psoriasis, one trial reported no development after 2019; no specific information about causes (the impact of the COVID-19 pandemic or others) behind this lack of development was found. Another phase I psoriasis trial stopped during recruitment. Finally. a third phase I trial [27] found that treatment with the ointment was generally safe and well tolerated. Thus, a clear link between the alleged properties of CBCPs and results from CTs with topical products was not found. The lack of clinical evidence regarding CBRMs for pain indications makes the claims made by CBCP retailers more concerning.

Most CTs focused on the short-term effects of cannabinoids, and no phase III CTs were found, so there is little information being gathered about the safety and efficacy of long-term treatments and their effectiveness compared to standard therapies. There is a need for high-quality clinical data on cannabinoids for medicinal purposes leading to safe and effective CBRMs. Progress is hindered by lack of funding and the complex and variable legislative restrictions including the sourcing of actives [56]. Additionally, research outcomes must be adequately communicated to policy makers, healthcare providers, and other stakeholders who will influence and enact policies, procedures, and laws related to cannabis use [56].

## 5. Conclusions

Despite the increased acceptance of *Cannabis sativa* and its derivatives as pharmacotherapeutic tools, only a limited number of regulator-approved cannabinoid-based medicines are available worldwide. In contrast, a wide range of non-regulator approved cannabis-based medicinal products are accessible to patients and consumers. Possibly, the strict and variable regulation of *Cannabis sativa* and related products, hinders the development of cannabis-based medicines, leaving a market gap filled with unregulated products that are potentially misleading regarding their therapeutic claims. Despite a growing interest in topical cannabinoids, only consumer products are currently available, some with unsubstantiated claims about “health” properties. Most clinical trials on cannabinoid-based products correspond to early development stages (phase I and II), illustrating the obstacles faced by sponsors. Research barriers include a lack of funding and legislative restrictions but also the unfair market competition posed by unregulated medicinal and consumer products. Well-designed legal structures and regulatory approaches are required to facilitate the development of cannabis-based medicines of suitable quality, safety, and efficacy, to which patients’ access is clearly enabled. Better health literacy regarding the use of cannabis-based medicines is essential for patients, healthcare professionals, and the public to make better-informed treatment choices.

## Figures and Tables

**Table 1:** Some significant regulatory events for cannabinoid-based medicinal products through the European Medicines Agency (EMA) and EU National Competent Authorities, the Food and Drug Administration (FDA), and the Therapeutic Goods Administration (TGA). Products listed as export-only by the TGA are presented in Table S3. Information gathered from the FDA [20], EMA [22,37], and TGA [24]. Last accessed on 10 December 2024. API: active pharmaceutical ingredient; CBD: cannabidiol; THC: delta-9-tetrahydrocannabinol. Nabiximols = extracts from *Cannabis sativa* L., containing THC, CBD, and lesser amounts of other cannabinoids. N/A = no authorised product found. See [4] for an exhaustive list of products authorised by EU member states.

Product Name Dosage Form	Regulatory Agency/ Regulatory Event/Status	Current Indication
**CBD**		
Epidyolex™ Oral solution	EMA First MA 2018 Orphan Medicine	Use as adjunctive therapy of seizures associated with Lennox–Gastaut syndrome (LGS) or Dravet syndrome (DS), in conjunction with clobazam, for patients 2 years of age and older. Use as adjunctive therapy of seizures associated with tuberous sclerosis complex (TSC) for patients 2 years of age and older.
Epidyolex™ Oral solution	FDA First approval 2018 NDA, Orphan; Priority	Treatment of seizures associated with Lennox–Gastaut syndrome, Dravet syndrome, or tuberous sclerosis complex in patients 1 year of age and older.
Epidyolex™ Oral solution	TGA ARTG start date 2020 Registered, Orphan; Priority	Use as adjunctive therapy of seizures associated with Lennox–Gastaut syndrome (LGS) or Dravet syndrome (DS) for patients 2 years of age and older.
N/A	EMA OD: 2015, 2016, 2017, 2021, 2022	2012: Treatment of complex regional pain syndrome. 2015: Treatment of perinatal asphyxia. 2016: Prevention of graft-versus-host disease. Treatment of graft-versus-host disease. 2017: Treatment of West syndrome. 2022: Treatment of epidermolysis bullosa; fragile X syndrome (FXS) of 22q11.2 deletion syndrome; and of epilepsy with myoclonic–atonic seizures.
**Nabiximol [THC + CBD] and Dronabinol + CBD**		
Sativex™ Oro-mucosal Spray	EU-NCAs and EMA (PIP), MHRA and AEMPS approval in 2010 through decentralised procedure. Further EU authorizations through mutual recognition procedure (2)	(MHRA label) Treatment for symptom improvement in adult patients with moderate-to-severe spasticity due to multiple sclerosis (MS) who have not responded adequately to other anti-spasticity medication and who demonstrate clinically significant improvement in spasticity related symptoms during an initial trial of therapy. Adults only.
**Nabiximols: HC and CBD**		
Sativex™ Oromucosal Spray	TGA ARTG Start date 2012 Registered	Treatment, for symptom improvement in patients with moderate-to-severe spasticity due to multiple sclerosis (MS) who have not responded adequately to other anti-spasticity medication and who demonstrate clinically significant improvement in spasticity related symptoms during an initial trial of therapy. Adults only.
**THC + CBD extracts from *Cannabis sativa* L**.		
N/A	EMA OD 2016	Treatment of glioma
**Dronabinol/CBD**		
N/A	EMA OD 10/2015 OD withdrawn 11/2015 at sponsor’s request.	Treatment of glioma.
**Dronabinol**		
Marinol Oral capsule	FDA First approval 1985 NDA, orphan *, priority.	Indicated in adults for the treatment of anorexia associated with weight loss in patients with AIDS * and of nausea and vomiting associated with cancer chemotherapy in patients who have failed to respond adequately to conventional antiemetic treatments. Notes: * OD indication: stimulation of appetite and prevention of weight loss in patients with a confirmed diagnosis of AIDS.
Marinol Therapeutic equivalents Oral capsule	FDA: Ascent Pharms Inc., Central Islip, NY, USA, ANDA 2020 Discontinued: Lannett Co Inc., Trevose, PA, USA, ANDA 2018; Hikma, ANDA 2014; Insys Therap, Chandler, AZ, USA, ANDA 2011; SVC Pharma, Coventry, UK, ANDA 2008.	Same as Marinol
Syndros	FDA First approval 2016 NDA Standard	Indicated in adults for the treatment of anorexia associated with weight loss in patients with AIDS and of nausea and vomiting associated with cancer chemotherapy in patients who have failed to respond adequately to conventional antiemetic treatments.
**Nabilone**		
N/A	EMA Negative decision on OD in 2011	OD requested: treatment of amyotrophic lateral sclerosis.
Cesamet	FDA First approval 1985/NDA Standard	Treatment of nausea and vomiting associated with cancer chemotherapy in patients who have failed to respond adequately to conventional antiemetic treatments.
**Curcuma longa**, **hemp seed oil**, **and piper nigrum**		
Pain relief Oral, soft capsule	TGA ARTG Start date 31 May 2023 Listed	Permitted Indications: Antioxidant/Reduce free radicals formed in the body, helps reduce/decrease free radical damage to body cells, anti-inflammatory/relieve inflammation, traditionally used in Ayurvedic medicine to anti-inflammatory/relieve inflammation, analgesic/Anodyne/relieve pain, traditionally used in Ayurvedic medicine to analgesic/Anodyne/relieve pain, decrease/reduce/relieve mild joint pain/soreness, traditionally used in Ayurvedic medicine to decrease/reduce/relieve symptoms of indigestion/dyspepsia. Indication Requirements: Label statement: If symptoms persist, talk to your health professional. Product presentation must only refer to mild joint symptoms. Product presentation must not imply or refer to bone disease or disorders, e.g., rheumatoid arthritis, juvenile arthritis, debilitating osteoarthritis, osteoporosis. Product presentation must not imply or refer to gastro oesophageal reflux disease. Warnings: Not to be taken by children under 2 years old. Not to be taken on the same day with other products containing hemp seed oil, including food sources.
**Hemp seed oil**		
Plant-Based Omega Oral, soft capsule	TGA ARTG start date: 21 May 2023 Listed	Permitted indications: antioxidant/reduces free radicals formed in the body, helps reduce/decrease free radical damage to body cells, maintains/supports general health and well-being, anti-inflammatory/relieves inflammation, decreases/reduces/relieves symptoms of mild eczema/dermatitis, maintains/supports skin health Indication requirements: label statement—If symptoms persist, talk to your health professional. Product presentation must only refer to mild eczema. Warnings: Not to be taken by children under 2 years old. Not to be taken on the same day with other products containing hemp seed oil, including food sources.

**Table 2:** Indications for which >50, 30–20, 19–10, 5–2 and 1 CT involving cannabinoids has been completed (Comp-CTs). The number in brackets indicates the number of trials for specific indications. Indications with the same number of completed CTs are listed alphabetically. Data gathered from the AdisInsight trial database; see Methods for search terms, last accessed on 11 December 2024. See Table S7 and Methods for inclusion and exclusion criteria.

**>30** **Comp-CTs**	Epilepsy [35], multiple sclerosis [31]
**30–20** **Comp-CTs**	Muscle spasticity [27], neuropathic pain [27], pain [24], cancer pain [21], Dravet syndrome [21], Lennox–Gastaut syndrome [21]
**19–10** **Comp-CTs**	Opioid-related disorders [16], infantile spasms [14], nausea and vomiting [13], seizures [13], Parkinson’s disease [12], schizophrenia [11], tuberous sclerosis [11]
**9–5** **Comp-CTs**	Anorexia [9], cerebral ischaemia [9], Alzheimer’s disease [6], chemotherapy-induced nausea and vomiting [6], pervasive child development disorders [6], back pain [5], diabetic neuropathies [5], fibromyalgia [5], glioblastoma [5], heroin-related disorders [5], Rett syndrome [5]
**4–1** **Comp-CTs**	Bladder dysfunction [4], Duchenne muscular dystrophy [4], ovarian cancer [4], Sturge–Weber syndrome [4], abdominal pain [3], acne [3], adenocarcinoma [3], blepharospasm [3], cystic fibrosis [3], type 2 diabetes mellitus [3], drug dependence [3], epidermolysis bullosa [3], fragile X syndrome [3], Gilles de la Tourette’s syndrome [3], graft-versus-host disease [3], postoperative pain [3], post-traumatic stress disorders [3], sleep apnoea syndrome [3], vascular dementia [2], atopic dermatitis [2], brain cancer [2], gastro-oesophageal reflux [2], Huntington’s disease [2], inflammation [2], insomnia [2], irritable bowel syndrome [2], psoriasis [2], ulcerative colitis [2]
**1** **Comp-CTs**	Acute pain, Angelman syndrome, anxiety and anxiety disorders, attention-deficit hyperactivity disorder, cardiovascular disorders, chest pain, CNS disorders, cognition disorders, Crohn’s disease, DiGeorge syndrome, drop attacks, drug-induced dyskinesia, duodenal ulcer, dyslipidaemias, dyspepsia, fatty liver, gastric ulcer, helicobacter infections, hypertension, liver cancer, memory disorders, metabolic disorders, migraine with aura, migraine without aura, motor neuron disease, neurogenic bladder, obesity, obsessive compulsive disorders, osteoarthritis, pancreatic cancer, panic disorders, peripheral nerve injuries, peptic ulcer, postnatal depression, postoperative nausea and vomiting, prostate cancer, rheumatoid arthritis, spinal cord disorders, staphylococcal infections, torticollis, trichotillomania, tuberculosis

**Table 3:** Indications for which 5, 4, 3, 2, and 1 discontinued clinical trial (Disc-CTs), suspended clinical trial (Susp-CT) and withdrawn clinical trial (With-CT) involving cannabinoids was found. Indications are listed alphabetically within each group. The number in brackets indicates the number of suspended and withdrawn trials for specific indications. Data gathered from the AdisInsight trial database, see Table S7 and methods for inclusion and exclusion criteria., last accessed on 11 December 2024.

**5 Disc-CTs**	Epilepsy
**4 Disc-CTs**	Muscle spasticity
**3 Disc-CTs**	Cancer pain, Prader–Willi syndrome
**2 Disc-CTs**	Neuropathic pain, post-traumatic stress disorders, Rett Syndrome
**1 Disc-CT**	Alzheimer’s disease, anorexia, anxiety and anxiety disorders, autoimmune hepatitis, back pain, cardiovascular disorders, chemotherapy-induced nausea and vomiting, drug dependence, dyslipidaemias, Gilles de la Tourette’s syndrome, glioblastoma, infantile spasms, inflammation, multiple sclerosis, nausea and vomiting, neurogenic inflammation, obesity, pervasive child development disorders, postherpetic neuralgia, postoperative nausea and vomiting, postoperative pain, schizophrenia, seizures
**Susp-CTs**	Cancer pain [2], multiple Sclerosis [1], diffuse scleroderma [1]
**With-CTs**	Acute pain [1], alcoholism [1], anxiety [1], cancer pain [2], chest pain [1], chemotherapy-induced damage [1], dementia [1], Dravet syndrome [1], endometriosis [1], Lennox–Gastaut syndrome [2], major depressive disorder [1], multiple sclerosis [1], muscle spasticity [1], nausea and vomiting [1], obsessive-compulsive disorders [1], pain [1], peripheral neuropathies [1], pervasive child development disorders [3], post-traumatic stress disorders [1], Prader–Willi syndrome [1], pruritus, wounds [1], rosacea [1], suicidal ideation [1]

**Table 4:** Trial name, indication, phase, status, sponsor, active ingredient, and identifier for CTs investigating topical cannabinoids for a variety of indications. Trials are grouped by indication and then listed by status (green: completed; grey: withdrawn; white: active/planning/recruiting). Unless mentioned otherwise, the names provided for the company and the sponsor provided were the same. Data gathered from the AdisInsight trials database (https://adisinsight.springer.com/, last accessed on 11 December 2024); see Methods for search terms and Table S7 for inclusion and exclusion criteria. N/A indicates that the CT identifier was no available from the Adisinsight database.

Trial Name	Indication	Phase	Status	Company/Sponsor	Agent/Actives	CT Identifier
Topical Cannabidiol (CBD) for the Treatment of Chemotherapy-Induced Peripheral Neuropathy: A Randomized Placebo-Controlled Pilot Trial	Peripheral neuropathies	II	Active, no longer recruiting	Mayo Clinic	Cannabidiol	NCT05388058
A phase I trial of PPP004, a topical cannabiniod based product, for the treatment of general neuropathic pain	Neuropathic pain	I	Planning	Tetra Bio Pharma	PPP-004	N/A
A Randomised, Double-Blind, Vehicle-Controlled Phase 2 Study of Topically Applied INM-755 (Cannabinol) Cream in Patients With Epidermolysis Bullosa	Epidermolysis bullosa	II	Completed	InMed Pharmaceuticals	Cannabidiol	NCT04908215
A Randomized, Double-Blind, Vehicle-Controlled, Phase I Study to Evaluate the Safety and Tolerability of Topically Applied INM-755 Cream on Epidermal Wounds in Healthy Volunteers	Epidermolysis bullosa	I	Completed	InMed Pharmaceuticals	Cannabidiol	NL8722
A Randomized, Double-Blind, Vehicle-Controlled, Phase I Study to Evaluate the Safety, Tolerability, and Pharmacokinetics of Topically Applied INM-755 Cream in Healthy Volunteers and to Study Suction Blisters as a Wound Healing Model.	Epidermolysis bullosa	I	Completed	InMed Pharmaceuticals	Cannabidiol	NL8269
Proof of concept trial of PPP004	Epidermolysis bullosa	II	Planning	Tetra Bio Pharma	PPP-004	N/A
First-in-human phase I trial of INM-750 (INM-755) in healthy volunteers	Epidermolysis bullosa	I	Planning	InMed Pharmaceuticals	Cannabidiol	N/A
A study to investigate INM 750 for the treatment of epidermolysis bullosa simplex	Epidermolysis bullosa	I	Planning	InMed Pharmaceuticals	Cannabidiol	N/A
A phase I/II study of INM-088 for the treatment of glaucoma	Glaucoma	I–II	Planning	InMed Pharmaceuticals	Cannabinol	N/A
A Randomized, Double-Blind, Vehicle-Controlled Study of the Safety, Tolerability and Efficacy of BTX 1204 in Patients With Moderate Atopic Dermatitis	Atopic dermatitis	II	Completed	Botanix Pharmaceuticals	Cannabidiol	NCT03824405
A Randomised, Double-Blind, Vehicle-Controlled Study of the Safety and Tolerability of BTX 1204 in Patients with Mild to Moderate Atopic Dermatitis	Atopic dermatitis	I	Completed	Botanix Pharmaceuticals	Cannabidiol	ACTRN12617001437358
A phase IIa, proof-of-concept study for examining the use of topical Cannabidiol CBD for the management of arthritis pain in the hand	Osteoarthritis	II	Active, no longer recruiting	Avecho Biotechnology	Cannabidiol	N/A
A Single Centre Observational Study to Assess whether a Topically Applied Cannabidiol Gel can Reduce Pain, Increase Grip Strength, or Improve Joint Functionality for Sufferers of Painful Osteoarthritis of the Hand	Musculoskeletal pain, osteoarthritis	I–II	Not yet recruiting	Avecho Biotechnology	Cannabidiol	ACTRN12621001512819p
A placebo control trial assessing topical Cannabidiol	Arthritis, Insomnia	II	Planning	Avecho Biotechnology	Cannabidiol	N/A
A Randomized, Double-Blind, Vehicle-Controlled Study to Evaluate the Safety and Efficacy of BTX 1503 in Patients With Moderate to Severe Acne Vulgaris	Acne vulgaris	II	Completed	Botanix Pharmaceuticals	Cannabidiol	NCT03573518
An Open-Label Study to Evaluate the Safety and Tolerability of BTX 1503 Solution in Patients with Acne Vulgaris	Acne vulgaris	I	Completed	Botanix Pharmaceuticals	Cannabidiol	ACTRN12617001127392
A Randomised, Double-Blind, Vehicle-Controlled Study of the Safety and Tolerability of BTX 1702 in Patients with Papulopustular Rosacea	Rosacea	I	Recruiting	Botanix Pharmaceuticals	Cannabidiol	ACTRN12621000689875
A Randomised, Double-Blind, Vehicle-Controlled Study of the Safety and Tolerability of Two Dosage Forms of BTX 1702 in Patients with Papulopustular Rosacea	Rosacea	I	Withdrawn prior to enrolment	Botanix Pharmaceuticals	Cannabidiol	ACTRN12620000184976
The Efficacy and Safety of 3% Cannabidiol (CBD) Cream in Patients With Epidermolysis Bullosa: A Phase II/III Trial	Pain, pruritus, and wounds	II–III	Withdrawn prior to enrolment	Avicanna	AVCN 583601	NCT04613102
Vehicle and Comparator-Controlled, Evaluator-blinded Trialto Evaluate the Safety and Anti-Psoriatic Efficacy of Topical Formulations of BTX 1308 in Subjects with Psoriasis Vulgaris in a Psoriasis Plaque Test	Plaque psoriasis	I	Completed	Botanix Pharmaceuticals	Cannabidiol; Betamethasone valerate	ACTRN12618001802291
A Phase I, Double Blind, Randomized, Placebo Controlled, Maximal Dose Study to Determine the Safety, Tolerability of Topical Cream Containing MGC (Medical Grade Cannabis) in Healthy Volunteers	Psoriasis	I	Completed	One World Cannabis	Nabiximols	NCT02976779
A 12-week safety study of Cannabinoid-Infused Topical Cream for the treatment of psoriasis and related skin conditions in healthy volunteers.	Psoriasis	I	Recruiting	One World Cannabis	Nabiximols	N/A
A Phase II efficacy study cannabinoid-based ointment for the treatment of Psoriasis: Psoriasis Study	Psoriasis	II	Planning	One World Cannabis	Nabiximols	N/A
A Pilot study to investigate the benefits of a cannabidiolic acid topical cream for the treatment of restless leg syndrome	Restless leg syndrome	I–II	Active, no longer recruiting	Synthonics	Cannabidiolic acid	NCT06570941

## Data Availability

Excel files providing the following raw data: Table S6. Cannabinoid-based clinical trials extracted from AdisInsight and Table S2. OD medicines listed on the EMA and FDA Repositories data is available in Supplementary Materials.

## Supplementary Materials

The following supporting information can be downloaded at: https://www.mdpi.com/article/10.3390/pharmaceutics17050635/s1. Supplementary material has been submitted separately as Table S1: Inclusion and exclusion criteria applied to screen literature collected from Web of Science; Table S2: OD medicines listed on the EMA and FDA repositories; Table S3: TGA export-only medicines and Excel files providing raw data; Table S4: Inclusion and exclusion criteria applied to screen data from the following regulatory depositories: EMA, FDA and ARTG; Table S5: Search terms for top-related topics; Table S6: Cannabinoid-based clinical trials extracted from AdisInsight; Table S7: Inclusion and exclusion criteria used when searching the AdisInsight database for clinical trial data.
