# Supplementary material for: The Regulatory Environment Surrounding Cannabis Medicines in the EU, the USA, and Australia
Source: Pharmaceutics. 2025 May 10;17(5):635. doi: 10.3390/pharmaceutics17050635 (PMC12115261; doi:10.3390/pharmaceutics17050635)
Supplement: Supplementary file 1 [file pharmaceutics-17-00635-s001.zip › Table S1.pdf]

Supplementary Information

Table S1. Inclusion and exclusion criteria applied to screen literature collected from Web of Science.

Criteria	Inclusion Criteria	Exclusion Criteria
**Topic**	• Articles discussing the regulation, prescription or access to cannabis-based medicinal products.	• Articles discussing recreational cannabis use and illicit markets.
**Article Type**	• Peer-reviewed journal articles, government reports and legal reviews.	• Opinion pieces, blog posts and non-peer reviewed sources.
**Publication Date**	• Time frame: 2019-2024	• Articles published before 2019.
**Language**	• Language: Written in English or translated to English.	• Studies were not in English language.
