# Supplementary material for: The Regulatory Environment Surrounding Cannabis Medicines in the EU, the USA, and Australia
Source: Pharmaceutics. 2025 May 10;17(5):635. doi: 10.3390/pharmaceutics17050635 (PMC12115261; doi:10.3390/pharmaceutics17050635)
Supplement: Supplementary file 1 [file pharmaceutics-17-00635-s001.zip › Table S3 Revised.pdf]

Supplementary information

Table S.3 TGA Export only medicines gathered by searching the ARTG data base (https://www.tga.gov.au/) using the terms dronabinol, THC, CBD, and nabilone and 31 December 2024 as latest start date(last access 24th April 2025). No permitted indications were included in the record for any of the products listed in this table.

ARTG name / Product name (when different)	Active	Sponsor and ARTG start date	Route and dosage form	Specific indications when described
T20:C5 LGP Classic / LGP CLASSIC 20:5	dronabinol 20 mg/mL THC 50mg/mL	Little Green Pharma Ltd 27/06/2019	Oral, oily liquid	There is growing evidence that medicinal cannabis can benefit patients in a variety of clinical settings. Based on this, the LGP CLASSIC oil range including 10:10 LGP CLASSIC, and 20:5 LGP CLASSIC as medicinal cannabis products, may have a role in medical conditions including: · Intractable chronic pain insufficiently responsive to other analgesics · Pain associated with spasticity in multiple sclerosis · Pain associated with cancer in palliative care · Cancer treatment-induced nausea and vomiting · Appetite loss · Insomnia · Post-traumatic stress disorder · Cachexia
10:10 LGP CLASSIC	CBD 10 mg/mL THC 10 mg/mL	Little Green Pharma Ltd 27/06/2019	Oral liquid	There is growing evidence that medicinal cannabis can benefit patients in a variety of clinical settings. Based on this, LGP CLASSIC oil range as medicinal cannabis products may have a role in medical conditions including: Intractable chronic pain insufficiently responsive to other analgesics, pain associated with spasticity in multiple sclerosis, pain associated with cancer in palliative care, cancer treatment-induced nausea and vomiting, appetite loss, insomnia·, post-traumatic stress disorder, cachexia.
20:5 LGP CLASSIC	CBD 5 mg/mL THC 20 mg/mL	Little Green Pharma Ltd 27/06/2019	Oral liquid	There is growing evidence that medicinal cannabis can benefit patients in a variety of clinical settings. Based on this, the LGP CLASSIC oil range including 10:10 LGP CLASSIC, and 20:5 LGP CLASSIC as medicinal cannabis products, may have a role in medical conditions including: Intractable chronic pain insufficiently responsive to other analgesics, pain associated with spasticity in multiple sclerosis, pain associated with cancer in palliative care, cancer treatment-induced nausea and vomiting; appetite loss, insomnia, post-traumatic stress disorder, cachexia
T10:C10 LGP CLASSIC	CBD 10 mg/mL THC 10 mg/mL	Little Green Pharma Ltd 27/06/2019	Oral, liquid	There is growing evidence that medicinal cannabis can benefit patients in a variety of clinical settings. Based on this, the LGP CLASSIC oil range as medicinal cannabis products may have a role in medical conditions including intractable chronic pain insufficiently responsive to other analgesics, pain associated with spasticity in multiple sclerosis, pain associated with cancer in palliative care, cancer treatment-induced nausea and vomiting, appetite loss, insomnia, post-traumatic stress disorder, cachexia
DEMECAN 16:01 FLORESTURA	CBD 0.01 g/g THC 0.16 g/g	Little Green Pharma Ltd 23/12/2020	Inhalation, dried herb	There is growing evidence that medicinal cannabis may have a role in several medical conditions. These include intractable chronic pain insufficiently responsive to other analgesics, pain associated with spasticity in multiple sclerosis (MS), pain associated with cancer in palliative care, nausea and vomiting caused by cancer treatment, appetite loss, sleep disturbance, post-traumatic stress disorder (PTSD), body wasting. Your doctor may have prescribed LGP medicinal cannabis dried flower for another reason
DEMECAN 18:01 FLORESTURA	CBD 0.01 g/g THC 0.18 g/g	Little Green Pharma Ltd 23/12/2020	Inhalation, dried herb	There is growing evidence that medicinal cannabis may have a role in several medical conditions. These include intractable chronic pain insufficiently responsive to other analgesics, pain associated with spasticity in multiple sclerosis (MS), pain associated with cancer in palliative care, nausea and vomiting caused by

ARTG name / Product name (when different)	Active	Sponsor and ARTG start date	Route and dosage form	Specific indications when described
				cancer treatment, appetite loss, sleep disturbance, post-traumatic stress disorder (PTSD), body wasting. Your doctor may have prescribed LGP medicinal cannabis dried flower for another reason.
DEMECAN 20:01 FLORESTURA	CBD 0.01 g/g<br>THC 0.2 g/g	Little Green Pharma Ltd<br>23/12/2020	Inhalation, dried herb	There is growing evidence that medicinal cannabis may have a role in several medical conditions. These include intractable chronic pain insufficiently responsive to other analgesics, pain associated with spasticity in multiple sclerosis (MS), pain associated with cancer in palliative care, nausea and vomiting caused by cancer treatment, appetite loss, sleep disturbance, post-traumatic stress disorder (PTSD), body wasting. Your doctor may have prescribed LGP<br>medicinal cannabis dried flower for another reason
NOIDECS T26	CBD 0<br>THC 26 mg/mL	Cannoperations Pty Ltd<br>25/11/2020	Oral, liquid	
NOIDECS T1:C100	CBD 100 mg/mL<br>THC 0	Cannoperations Pty Ltd<br>9/12/2020	Oral, liquid	
NOIDECS T10:C15	CBD 15 mg/mL<br>THC 10 mg/mL	Cannoperations Pty Ltd<br>25/11/2020	Oral, liquid	
Medleaf High CBD Oil	CBD 100 mg/mL	Epsilon Pharm Pty Ltd<br>9/11/2020	Oral, liquid	Chronic Pain, Anxiety, Insomnia
Medleaf Pure CBD Oil	CBD 100 mg/mL	Epsilon Pharm Pty Ltd<br>5/11/2020	Oral, liquid	Chronic Pain, Anxiety, Insomnia
Medleaf Balanced THC/CBD Oil	CBD 25 mg/mL<br>THC 25 mg/mL	Epsilon Pharm Pty Ltd<br>5/11/2020	Oral, liquid	Chronic Pain, Anxiety, Insomnia
Althea THC5: CBD10	CBD<br>THC	Althea Company Pty Ltd<br>25/11/2020	Oral, liquid	Cancellation by Sponsor 28/06/2024<br>Cancelled under Section 30(1) (c) of the Act
Althea THC2: CBD25	CBD<br>THC	Althea Company Pty Ltd<br>19/11/2020	Oral, liquid	
Althea THC10: CBD12	CBD<br>THC	Althea Company Pty Ltd<br>25/11/2020	Oral, liquid	
Althea CBD12: THC10	CBD<br>THC	Althea Company Pty Ltd<br>22/09/2020	Oral, liquid	
Althea CBD10: THC5	CBD<br>THC	Althea Company Pty Ltd<br>22/09/2020	Oral, liquid	
Althea CBD25: THC2	CBD<br>THC	Althea Company Pty Ltd<br>22/09/2020	Oral, liquid	
Althea THC20: CBD1	CBD<br>THC	Althea Company Pty Ltd<br>22/09/2020	Oral, liquid	
1:20 LGP CLASSIC	CBD 20 mg/mL<br>THC 1 mg/mL	Little Green Pharma Ltd<br>3/02/2020	Oral, liquid	There is growing evidence that medicinal cannabis can benefit patients in a variety of clinical settings. Based on this, the LGP CLASSIC oil range including 1:20 LGP CLASSIC, as a medicinal cannabis product, may have a role in medical conditions including: intractable epileptic seizures in adults and children, intractable chronic pain insufficiently responsive to other analgesics, pain associated with spasticity in

ARTG name / Product name (when different)	Active	Sponsor and ARTG start date	Route and dosage form	Specific indications when described
				multiple sclerosis, pain associated with cancer in palliative care, cancer treatment-induced nausea and vomiting, appetite loss, insomnia, post-traumatic stress disorder, cachexia.
CBD 50 LGP CLASSIC	CBD 50 mg/mL	Little Green Pharma Ltd 16/09/2020	Oral, liquid	Intractable chronic pain insufficiently responsive to other analgesics, pain associated with spasticity in multiple sclerosis (MS), pain associated with cancer in palliative care, cancer treatment-induced nausea and vomiting, appetite loss, insomnia, post-traumatic stress disorder (PTSD), cachexia, anxiety, refractory epilepsy
T1:C20 LGP CLASSIC	CBD 20 mg/mL<br>THC 1 mg/mL	Little Green Pharma Ltd 20/02/2020	Oral, liquid	There is growing evidence that medicinal cannabis can benefit patients in a variety of clinical settings. Based on this, the LGP CLASSIC oil range including 10:10 LGP CLASSIC, and 20:5 LGP CLASSIC as medicinal cannabis products, may have a role in medical conditions including:behavioural issues associated with autism, intractable epileptic seizures in adults and children, intractable chronic pain insufficiently responsive to other analgesics, pain<br>associated with spasticity in multiple sclerosis, pain associated with cancer in palliative care, cancer treatment-induced nausea and vomiting, appetite loss, insomnia, post-traumatic stress disorder, cachexia
Canndeo Canada CBD 25 |CBD Oil	CBD 25 mg/mL	Epsilon Pharm Pty Ltd 29/07/2020	Oral, liquid	Insomnia (particularly in improving sleep quality), anxiety disorder, acronic inflammatory pain, depression
iuvo THC26	CBD 0.001 g/g<br>THC 0.027 g/g	Cannoperations Py Ltd 4/05/2021	Oral, liquid	
Cannabis Oil - CBD 100	CBD 100 mg/mL	ABC Can Pty Ltd 12/09/2021	Oral, oil	
Xativa 12.5mg	CBD 12.5 mg	iX Biopharma Pty Ltd 22/04/2021	Sublingual , wafer	
Xativa 25 mg	CBD 25 mg	iX Biopharma Pty Ltd 22/04/2021	Sublingual , wafer	
10:50 Cannabisextrakt	CBD 10 mg/mL<br>THC 50mg/mL	OneLife Labs Pty Ltd 8/09/2021	Oral, liquid	
25:25 Cannabisextrakt	CBD 25 mg/mL<br>THC 25 mg/mL	OneLife Labs Pty Ltd 8/09/2021	Oral, liquid	
10:10 Cannabisextrakt	CBD 10 mg/mL<br>THC 10 mg/mL	OneLife Labs Pty Ltd 30/08/2021	Oral, liquid	
Turkken 10:10	CBD 10 mg/mL<br>THC 10 mg/mL	Health House International Pty Ltd 1/08/2021	Buccal, solution	Chronic pain, drug resistant epilepsy, nausea in cancer related chemotherapy, multiple sclerosis
Adjupharm THC 10:10 CBD	CBD 10 mg/mL<br>THC 10 mg/mL	OneLife Labs Pty Ltd 15/09/2021	Oral, liquid	
Adjupharm THC 25:1 CBD	CBD 0<br>THC 25 mg/mL	OneLife Labs Pty Ltd 15/09/2021	Oral, liquid	
DEMECAN 22:01 FLORESTURA	CBD 0.01 g/g<br>THC 0.22 g/g	Little Green Pharma Pty Ltd 25/03/2021	Inhalation, herb dried	There is growing evidence that medicinal cannabis may have a role in several medical conditions. These include intractable chronic pain insufficiently responsive to other analgesics, pain associated with spasticity in multiple sclerosis (MS), pain associated with cancer in palliative care, nausea and vomiting caused by

ARTG name / Product name (when different)	Active	Sponsor and ARTG start date	Route and dosage form	Specific indications when described
				cancer treatment, appetite loss, sleep disturbance, post-traumatic stress disorder (PTSD), body wasting. Your doctor may have prescribed LGP medicinal cannabis dried flower for another reason
DEMECAN 24:01 FLORESTURA	CBD 0.01 g/g<br>THC 0.24 g/g	Little Green Pharma Pty Ltd<br>25/03/2021	Inhalation, herb dried	There is growing evidence that medicinal cannabis may have a role in several medical conditions. These include intractable chronic pain insufficiently responsive to other analgesics, pain associated with spasticity in multiple sclerosis (MS), pain associated with cancer in palliative care, nausea and vomiting caused by cancer treatment, appetite loss, sleep disturbance, post-traumatic stress disorder (PTSD), body wasting. Your doctor may have prescribed LGP medicinal cannabis dried flower for another reason
Lumir UK CBD 50	CBD1.587 mL<br>THC 0.033 mL	Canim Australia Pty Ltd<br>20/08/2021	Oral, oil	
Lumir UK THC 27	CBD 0.033 mL<br>THC 0.858 mL	Canim Australia Pty Ltd<br>20/08/2021	Oral, oil	
Cann Group THC26	CBD 0.001 g/g<br>THC 0.858 g/g	Cannoperations Pty Ltd<br>4/05/2021	Oral, liquid	
MediPharm Labs Extract 10:10 THC:CBD	CBD 10 mg/mL<br>THC 10 mg/mL	OneLife Labs Pty Ltd<br>16/11/2021	Oral, liquid	
MediPharm Labs Extract 12.5:12.5 THC:CBD	CBD 12.5 mg/mL<br>THC 12.5 mg/mL	OneLife Labs Pty Ltd<br>16/11/2021	Oral, liquid	
MediPharm Labs Extract 25:1 THC:CBD	CBD 0<br>THC 25 mg/mL	OneLife Labs Pty Ltd<br>16/11/2021	Oral, liquid	
MediPharm Labs Extract 25:25 THC:CBD	CBD 25 mg/mL<br>THC 25 mg/mL	OneLife Labs Pty Ltd<br>16/11/2021	Oral, liquid	
MediPharm Labs Extract 1:30 THC:CBD	CBD 30 mg/mL<br>THC 0	OneLife Labs Pty Ltd<br>6/12/2021	Oral, liquid	
MediPharm Labs 20:1 THC	CBD 0<br>THC 20 mg/mL	OneLife Labs Pty Ltd<br>30/6/2021	Oral, liquid	
MediPharm Labs 50:1 THC	CBD 0<br>THC 50 mg/mL	OneLife Labs Pty Ltd<br>30/6/2021	Oral, liquid	
MediPharm Labs 10:1 THC	CBD 0<br>THC 10 mg/mL	OneLife Labs Pty Ltd<br>6/05/2021	Oral, liquid	
OneLife Labs 30:1 THC	CBD 0<br>THC 30 mg/mL	OneLife Labs Pty Ltd	Oral, liquid	
MediPharm Labs 10:15 THC:CBD	CBD 15 mg/mL<br>THC 10 mg/mL	OneLife Labs Pty Ltd<br>6/05/2021	Oral, liquid	
MediPharm Labs 20:1 CBD	CBD 20 mg/mL<br>THC 0	OneLife Labs Pty Ltd<br>6/05/2021	Oral, liquid	
MediPharm Labs 50:2 CBD	CBD 50 mg/mL<br>THC 0	OneLife Labs Pty Ltd<br>6/05/2021	Oral, liquid	
MediPharm Labs 25:1 CBD	CBD 25 mg/mL<br>THC 0	OneLife Labs Pty Ltd<br>25/03/2021	Oral, liquid	

ARTG name / Product name (when different)	Active	Sponsor and ARTG start date	Route and dosage form	Specific indications when described
MediPharm Labs 25:1 THC	CBD 0<br>THC 25 mg/mL	OneLife Labs Pty Ltd<br>25/03/2021	Oral, liquid	
MediPharm Labs 12.5:12.5 Balanced	CBD 12.5 mg/mL<br>THC 12.5 mg/mL	OneLife Labs Pty Ltd<br>6/05/2021	Oral, liquid	
OneLife Labs 20:20 Balanced	CBD 20 mg/mL<br>THC 20 mg/mL	OneLife Labs Pty Ltd<br>23/04/2021	Oral, liquid	
MediPharm Labs 10:10 Balanced	CBD 10 mg/mL<br>THC 10mg/mL	OneLife Labs Pty Ltd<br>25/03/2021	Oral, liquid	
MediPharm Labs AU Balanced 10:10	CBD 10 mg/mL<br>THC 10mg/mL	OneLife Labs Pty Ltd<br>28/06/2021	Oral, liquid	
MediPharm Labs AU 25:1 CBD	CBD 25 mg/mL<br>THC 0	OneLife Labs Pty Ltd<br>17/06/2021	Oral, liquid	
MediPharm Labs THC 25:1 CBD	CBD 0<br>THC 25 mg/mL	OneLife Labs Pty Ltd<br>17/06/2021	Oral, liquid	
Lumir Cannabis Extrakt Balanced 10:10	CBD 0.318 mL<br>THC 0.318 mL	Cannim Australia Pty Ltd<br>20/08/2021	Oral, oil	
Lumir Cannabis Extrakt THC 25	CBD 0.033 mL<br>THC 0.795 mL	Cannim Australia Pty Ltd<br>20/08/2021	Oral, oil	
Lumir UK 10 10 Balanced<br>Lumir UK 10 10	CBD 0.318 mL<br>THC 0.318 mL	Cannim Australia Pty Ltd<br>20/08/2021	Oral, oil	
iuvo Balance THC10:CBD15	CBD 0.016 g/g<br>THC 0.01 g/g	Cannoperatios Pty Ltd<br>4/05/2021	Oral, liquid	
Cann Group Balance THC10:CBD15	CBD 0.016 g/g<br>THC 0.01 g/g	Cannoperatios Pty Ltd<br>4/05/2021	Oral, liquid	
Nanadol	CBD 9.62 mg/mL<br>THC 9.62 mg/mL	Medlab Clinical Ltd<br>17/06/2022	Buccal, spray, solution	
CBD100, 30 mL	CBD 100 mg/mL	IDT Australia Ltd<br>23/09/2022	Oral, liquid	
CBD100, 50 mL	CBD 100 mg/mL	IDT Australia LTD<br>23/09/2022	Oral, liquid	
1:100 LGP CLASSIC	CBD 100 mg/mL<br>THC1 mg/mL	Little Green Pharma Pty Ltd<br>2/03/2022	Oral, liquid	There is growing evidence that medicinal cannabis may have a role in treating the symptoms of many medical conditions. These include chronic pain, spasticity and pain in multiple sclerosis (MS), pain associated with cancer in palliative care, nausea and vomiting caused by cancer treatment, appetite loss, sleep disturbance, post-traumatic stress disorder (PTSD), body wasting, anxiety and drug-resistant epilepsy
MediPharm Labs 10:100 THC:CBD	CBD 100 mg/mL<br>THC 10 mg/mL	OneLife Labs Pty Ltd<br>11/05/2022	Oral, liquid	

ARTG name / Product name (when different)	Active	Sponsor and ARTG start date	Route and dosage form	Specific indications when described
MediPharm Labs 30:30 Balanced	CBD 30 mg/mL THC 30 mg/mL	OneLife Labs Pty Ltd 11/05/2022	Oral, liquid	
Adjupharm THC 1:20 CBD	CBD 20 mg/mL THC 0	OneLife Labs Pty Ltd 20/01/2022	Oral, liquid	
Adjupharm THC 2:50 CBD	CBD 50 mg/mL THC 2 mg/mL	OneLife Labs Pty Ltd 20/01/2022	Oral, liquid	
Adjupharm THC 4:100 CBD	CBD 100 mg/mL THC 4 mg/mL	OneLife Labs Pty Ltd 20/01/2022	Oral, liquid	
20:1 Medicinal Cannabis Oil	CBD 1 mg/mL THC 20 mg/mL	ECS Botanics MC Pty Ltd 6/01/ 2022	Oral, oil	
THC 18% Flower 10g	THC 180 mg/g	Tasmanian Botanics Pty Ltd 10/10/2022	Inhalation, dried herb	
Lumir CBD 100 Full Spectrum - Cannabis Oil (1:100 THC/CBD)	CBD 7.362 mL THC 0.264 mL	Cannim Australia Pty Ltd 28/03/2022	Oral, liquid	
Lumir 25:25 Balanced Cannabis Oil (THC 25 mg/mL, CBD 25mg/mL)	CBD 0.81 mL THC 0.81 mL	Cannim Australia Pty Ltd 28/03/2022	Oral, liquid	
Lumir 12.5:12.5 Balanced Cannabis Oil 30mL (THC 12.5 mg/mL, CBD 12.5mg/mL)	CBD 0.35 mL THC 0.36 mL	Cannim Australia Pty Ltd 28/03/2022	Oral, liquid	
Lumir THC 25 Cannabis Oil (25mg/mL THC, less than or equal to 1mg/mL CBD)	CBD 0.003 mL THC 0.73 mL	Cannim Australia Pty Ltd 28/03/2022	CBD THC	
10:10 Medicinal Cannabis Oil 30mL	CBD 10 mg/mL THC 10 mg/mL	ECS Botanics Pty Ltd 16/03/2023	Oral, oil	
Lumir UK CC8/CA - Dried Cannabis Flower (THC 22%, CBD less than 1%)	THC 220 mg/g	Cannim Australia Pty Ltd 3/07/2023	Inhalation, herb dried	
Xativa Sleep 20mg/5mg	CBD 20 mg melatonin 5 mg	iXBiopharma Pty Ltd 06/12/2023	Sublingual , wafer	
Xativa 50mg	CBD 50 mg	iXBiopharma Pty Ltd 5 /04/2023	Sublingual , wafer	
Circle Balance 20:12 Cannabis Oil - 30ml	CBD 20 mg/mL THC 12 mg/mL	Montu Group Pty Ltd 11/08/2023	Oral, oil	

ARTG name / Product name (when different)	Active	Sponsor and ARTG start date	Route and dosage form	Specific indications when described
Circle Balance 25:25 Cannabis Oil - 30ml	CBD 25 mg/mL<br>THC 25 mg/mL	Montu Group Pty Ltd<br>11/08/2023	Oral, oil	
Circle Balance 10:10 Cannabis Oil - 30ml	CBD 10 mg/mL<br>THC 1 mg/mL	Montu Group Pty Ltd<br>01/09/2023	Oral, oil	
Circle THC 20 Cannabis Oil - 30ml	CBD 1 mg/mL<br>THC 20 mg/mL	Montu Group Pty Ltd<br>01/09/2023	Oral, oil	
Circle CBD Pure 100 Cannabis Oil - 30ml	CBD 100 mg/mL<br>THC 1 mg/mL	Montu Group Pty Ltd<br>01/09/2023	Oral, oil	
Circle CBD 20 Cannabis Oil - 30mL	CBD 20 mg/mL<br>THC 3 mg/mL	Montu Group Pty Ltd<br>11/08/2023	Oral, oil	
NOIDECS T25:C25	CBD 25 mg/mL<br>THC 25 mg/mL	Cannoperations Pty Ltd16/03/2023	Oral, oral liquid	
Cybis 10:25	CBD 25 mg/mL<br>THC 10 mg/mL	Cymra Life Sciences Ltd<br>24/01/2023	Oral, oral liquid	Cybis 10:25 is a medicinal cannabis formulation in conformance with the requirements set out in TGO 93. It is indicated for medical conditions as specified by the authorised prescriber through the TGA Special Access Scheme (Category B). It is not registered on the ARTG and as such no specific indications have been approved for the product
Rua Kia Kaha 26/1	CBD 0<br>THC 0.26 g/g	Cannoperations Pty Ltd<br>25/01/2023	Oral, herb dried	
Rua Kia Kaha 29/1	CBD 0<br>THC 0.29 g/g	Cannoperations Pty Ltd<br>25/01/2023	Oral, herb dried	
Strawberry Kush Dried Cannabis Flower	THC 255 mg/g	ECS Botanics MC Pty Ltd<br>8/05/2023	Oral, herb dried	
Murray Sherbet Dried Cannabis Flower	THC 20 mg/g	ECS Botanics MC Pty Ltd<br>12/05/2023	Oral, herb dried	
Murray Milkshake Dried Cannabis Flower - Bulk	THC 230 mg/g	ECS Botanics MC Pty Ltd<br>30/01/2023	Oral, herb dried	
HummingBud Sandy Bay 24% THC	THC 230 mg/g	Cannim Australia Pty Ltd<br>14/03/2023	Inhalation, herb dried	
Cannabis Oil - Full Spectrum CBD 50	CBD 50 mg/mL	ABC Can Pty Ltd<br>26/02/2024	Oral, oil	
Cannabis Oil - Full Spectrum THC:CBD 10:10	CBD 10 mg/mL<br>THC 10 mg/mL	ABC Can Pty Ltd<br>26/02/2024	Oral, oil	
Cannabis Oil - Full Spectrum THC 30	THC 30 mg/mL	ABC Can Pty Ltd<br>26/02/2024	Oral, oil	
Rua Puawai THC26	CBD 10 mg/g<br>THC 260 mg/g	Rua Bioscience Australia Pty Ltd<br>01/02/2024	Inhalation, herb dried	
Cannabis Oil - Full Spectrum THC 30	THC 30 mg/mL	ABC Can Pty Ltd<br>26/02/2024	Oral, oil	

ARTG name / Product name (when different)	Active	Sponsor and ARTG start date	Route and dosage form	Specific indications when described
Rua Hinu CBD100	CBD 100 mg/mL<br>THC 1 mg/mL	Rua Bioscience Australia Pty Ltd<br>03/06/2024	Oral, liquid	
Rua Puawai THC20	CBD 10 mg/g<br>THC 200 mg/g	Rua Bioscience Australia Pty Ltd<br>03/06/2024	Inhalation, herb dried	
Rua Puawai THC25	CBD 10 mg/g<br>THC 250 mg/g	Rua Bioscience Australia Pty Ltd<br>03/06/2024	Inhalation, herb dried	
Rua Hinu T10:C15	CBD 15 mg/g<br>THC 10 mg/g	Rua Bioscience Australia Pty Ltd<br>03/06/2024	Oral, liquid	
Rua Hinu T25:C25	CBD 25 mg/g<br>THC 25 mg/g	Rua Bioscience Australia Pty Ltd<br>03/06/2024	Oral, liquid	
Superseed T26	CBD 10 mg/g<br>THC 260 mg/g	Canadian Craft Cannabis Company Pty Ltd<br>27/11/2024	Inhalation, herb dried	
Superseed T27	CBD 10 mg/g<br>THC 270 mg/g	Canadian Craft Cannabis Company Pty Ltd<br>27/11/2024	Inhalation, herb dried	
Mystery Mountain T23	CBD10 mg/g<br>THC 230 mg/g	Canadian Craft Cannabis Company Pty Ltd<br>27/11/2024	Inhalation, herb dried	
Mystery Mountain T24	CBD 10 mg/g<br>THC 240 mg/g	Canadian Craft Cannabis Company Pty Ltd<br>27/11/2024	Inhalation, herb dried	
Spirit Bear T23	CBD 10 mg/g<br>THC 230 mg/g	Canadian Craft Cannabis Company Pty Ltd<br>27/11/2024	Inhalation, herb dried	
Spirit Bear T25	CBD 10 mg/g<br>THC 250 mg/g	Canadian Craft Cannabis Company Pty Ltd<br>27/11/2024	Inhalation, herb dried	
CBD 200 LGP PURE	CBD 200 mg/mL	Little Green Pharma Ltd<br>26/11/2024	Oral, liquid	
Spirit Bear T9/C13	CBD 130 mg/g<br>THC 90 mg/g	Canadian Craft Cannabis Company Pty Ltd<br>5/12/2024	Inhalation, herb dried	

ARTG name / Product name (when different)	Active	Sponsor and ARTG start date	Route and dosage form	Specific indications when described
Spirit Bear T10/C12	CBD 120 mg/g<br>THC 100 mg/g	Canadian Craft Cannabis Company Pty Ltd<br>5/12/2024	Inhalation, herb dried	
Pink Sherbet	THC 200 mg/g	ECS Botanics MC Pty Ltd<br>23/12/2024	Inhalation, herb dried	
Medigrowth Balanced (12.5:12.5 mg/mL THC: CBD) Medicinal Cannabis Oil - 30ml	CBD 12.5 mg/mL<br>THC 12.5 mg/mL	Medigrowth Trading Australia Pty Ltd<br>24/12/2024	Oral, oil	
Medigrowth THC50 mg/mL (50:1 mg/mL THC:CBD) Medicinal Cannabis Oil - 30mL	CBD 1 mg/mL<br>THC 50 mg/mL	Medigrowth Trading Australia Pty Ltd<br>24/12/2024	Oral, oil	
Medigrowth CBD100 mg/mL (0:100 mg/mL THC:CBD) Medicinal Cannabis Oil - 30mL	CBD 100 mg/mL	Medigrowth Trading Australia Pty Ltd<br>24/12/2024	Oral, oil	
