# Supplementary material for: The Regulatory Environment Surrounding Cannabis Medicines in the EU, the USA, and Australia
Source: Pharmaceutics. 2025 May 10;17(5):635. doi: 10.3390/pharmaceutics17050635 (PMC12115261; doi:10.3390/pharmaceutics17050635)
Supplement: Supplementary file 1 [file pharmaceutics-17-00635-s001.zip › Table S4..pdf]

**Supplementary Information**

**Table S.4** Inclusion and exclusion criteria applied to screen data from the following regulatory depositories: EMA, FDA and ARTG.

| Database | Inclusion Criteria | Exclusion Criteria |
|---|---|---|
| **EMA** | • Product category: Human medicines<br>• Active Pharmaceutical Ingredient (API) must be: cannabidiol, nabiximol, tetrahydrocannabinol, dronabinol and cannabis<br>• Paediatric Investigation Plans (PIP)<br>• European Public Assessment Reports (EPARs) | • Products from the categories: corporate, herbal and veterinary<br>• Active Pharmaceutical Ingredients not related to cannabinoids |
| **Drugs@FDA** | • Product category: drugs<br>• Active Pharmaceutical Ingredient (API) must be: cannabidiol, nabiximol, tetrahydrocannabinol, dronabinol and cannabis<br>• FDA reviews, letters and product labels | • Product category: animal & veterinary, cosmetics, dietary supplements, food & beverages, medical devices and tobacco<br>• Active Pharmaceutical Ingredients not related to cannabinoids |
| **ARTG** | • Product type: medicine<br>• Active Pharmaceutical ingredient (API): dronabinol, tetrahydrocannabinol, nabiximol, cannabidiol, hemp, nabilone<br>• Public summaries, patient information leaflets and product information | • Product type: Device or biological<br>• Active Pharmaceutical Ingredients not related to cannabinoids<br>• |
