# Supplementary material for: The Regulatory Environment Surrounding Cannabis Medicines in the EU, the USA, and Australia
Source: Pharmaceutics. 2025 May 10;17(5):635. doi: 10.3390/pharmaceutics17050635 (PMC12115261; doi:10.3390/pharmaceutics17050635)
Supplement: Supplementary file 1 [file pharmaceutics-17-00635-s001.zip › Table S7...pdf]

Supplementary Information

Table S.6 Inclusion and exclusion criteria applied to screen data from AdisInsight Database

Criteria	Inclusion Criteria	Exclusion Criteria
**Drug Class**	• Primary drug for trial: cannabinoids	• Trials not involving cannabinoids (plant-derived or synthetic) as the primary drug<br>• Trials using endocannabinoid mimetics as the primary drug
